# Supplementary material for: Quantitative assessment of multiple fish species around artificial reefs combining environmental DNA metabarcoding and acoustic survey
Source: Sci Rep. 2021 Sep 30;11:19477. doi: 10.1038/s41598-021-98926-5 (PMC8484675; doi:10.1038/s41598-021-98926-5)
Supplement: Supplementary file 1 — Supplementary Information. [file 41598_2021_98926_MOESM1_ESM.docx]

Supplementary materials

**Title**

Quantitative assessment of multiple fish species around artificial reefs combining environmental DNA metabarcoding and acoustic survey

**Authors**

Masaaki Sato^1*^, Nariaki Inoue^1^, Ryogen Nambu^1,2^, Naoki Furuichi^1^, Tomohito Imaizumi^1^, and Masayuki Ushio^3,4^

^1^Fisheries Engineering Division, Fisheries Technology Institute, Japan Fisheries Research and Education Agency (FRA), Kamisu, Ibaraki, Japan

* Correspondence (E-mail: msato22@affrc.go.jp)

^2^Present address: Fisheries Division, Japan International Research Center for Agricultural Sciences (JIRCAS), Tsukuba, Ibaraki, Japan

^3^Hakubi Center, Kyoto University, Kyoto 606-8501 Japan

^4^Center for Ecological Research, Kyoto University, Otsu, Shiga 520-2113 Japan


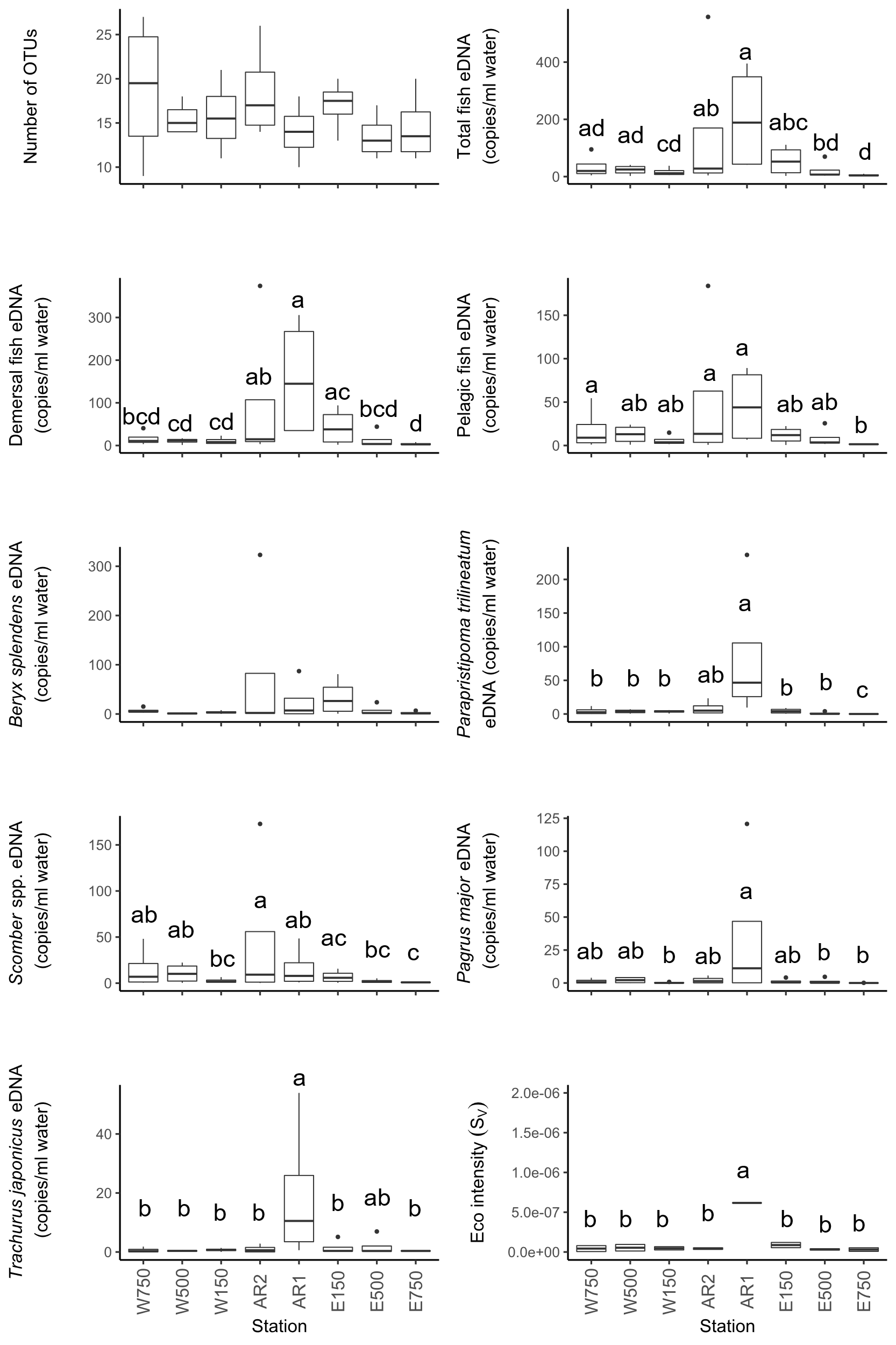


Figure S1 Box plots indicate number of OTUs, eDNA copies of total fish, demersal fish, pelagic fish, *Beryx splenden*s, *Parapristipoma trilineatum*, *Scomber spp.*, *Pagrus major*, and *Trachurus japonicus*, and eco intensity at each station. Different lowercase letters represent statistically significant differences (P < 0.05; Tukey tests).

Figure S2 Vertical profiles of seawater temperature (T, °C, solid line) and salinity (S, dashed line). The gray and black shading indicate the depth range of water sampling and bottom topography, respectively.

Table S1 Details of the eight sampling stations for fish eDNA survey on May 23, 2018

Table S2. Average eDNA copy numbers of each fish species in study stations and depth layers at Tateyama Bay. *Fish catch (kg) by a set net near study site in May 2018.

Table S3. Species list of fish catch (kg) by a set net near study site in May 2018.

Table S4. Results of likelihood ratio tests examining variation in number of OTUs, eDNA copies of fishes, and eco intensity among sampling stations.

Table S5. Summary results of Gamma or Gamma hurdle models (Null model: model with intercept only; minimum BIC model) for each response variable of eDNA copy numbers based on automated model selection.

Table S6. Sequences of MiFish primer forward and reverse regions extracted from MitoFish database (http://mitofish.aori.u-tokyo.ac.jp/) for dominant OTUs.
